# Antenatal screening for TB disease: a systematic review and meta-analysis

**DOI:** 10.5588/ijtldopen.25.0105

**Published:** 2025-06-13

**Authors:** A.J. Morton, N. Meagher, G. Tonkin-Hill, J.T. Denholm, R.I. Zahroh, S.J. Dunstan

**Affiliations:** ^1^ Department of Infectious Diseases, Melbourne Medical School, The University of Melbourne, at the Peter Doherty Institute for Infection and Immunity, 792 Elizabeth St, Melbourne, Victoria, Australia;; ^2^ Victorian Tuberculosis Program, Melbourne Health, at the Peter Doherty Institute for Infection and Immunity, Level 5, 792 Elizabeth St, Melbourne, Victoria, Australia;; ^3^ Victorian Infectious Diseases Service, Melbourne Health, Parkville, at the Peter Doherty Institute for Infection and Immunity, 792 Elizabeth St, Melbourne, Victoria, Australia;; ^4^ Gender and Women’s Health Unit, Nossal Institute for Global Health, Melbourne School of Population and Global Health, The University of Melbourne, Grattan Street, Parkville, Victoria, Australia.

**Keywords:** pregnancy, obstetrics, tuberculosis, screening, TST, IGRA

## Abstract

**OBJECTIVES:**

TB disease during pregnancy is associated with poor maternal and neonatal outcomes, and is a leading non-obstetric cause of maternal death. However, optimal detection strategies remain uncertain. We aimed to identify the optimal screening approach for TB disease in pregnant women.

**METHODS:**

We searched Ovid MEDLINE, Embase + Embase Classic, Web of Science, and CENTRAL to identify antenatal screening studies for TB disease. The yield, number needed to screen (NNS), and positive predictive value (PPV) were calculated for each method. Pooled estimates were generated using random-effects meta-analyses. Narrative synthesis was conducted to summarise secondary outcomes.

**RESULTS:**

We included 33 studies. Pooled yield for symptom screening (SS) was 7.26 [95% CI: 0.70, 19.25] cases per 1,000 versus 5.12 [95% CI: 0.79, 12.39] for TST/IGRA. NNS was 138 [95% CI: 51.95, 1,428.57] for SS versus 1,667 [95% CI: 537.63, 1,000,000] for TST/IGRA. SS pooled PPV was 3.85% [95% CI: 1.23–7.57%], and <0.01% [95% CI: <0.01–0.05%] for TST/IGRA. Narrative synthesis indicated antenatal SS is low-cost, feasible, and acceptable but poorly implemented.

**CONCLUSION:**

In pregnancy, symptom screening demonstrates highest yield and lowest NNS, is low-cost, feasible and acceptable. While currently optimal, the low PPV underscores the need for TB screening tools tailored to pregnant populations.

In 2022, an estimated 10.6 million people developed TB globally, with 1.3 million deaths.^1^ It is estimated that over 200,000 of these TB cases were among pregnant women.^2^ TB disease during pregnancy is associated with poor maternal and neonatal outcomes, including an increased risk of preterm birth, low birth weight and fetal death.^2-4^ Additionally, TB is a leading non-obstetric cause of maternal death.^4^ Pregnant and postpartum women are up to twice as likely to develop TB disease compared to their nonpregnant counterparts,^5,6^ which may be attributed to factors such as immune system changes during pregnancy.^7–9^ Although WHO strongly advocates for antenatal screening for TB when community prevalence exceeds 100 cases per 100,000 individuals,^10^ in practice, antenatal screening is infrequently conducted, primarily due to the absence of specific guidelines delineating optimal methods.^11^ The current WHO-recommended symptom screen involves questioning individuals about the presence of one or more of cough, weight loss, fever and night sweats.^12^ However, this approach has proven ineffective for pregnant women^11,13^ and the optimal approach to screening in pregnancy remains unclear, limiting the effective identification of TB disease within this demographic.

Considering the need for effective screening for TB in pregnancy, we aimed to conduct a systematic review and meta-analysis of the published literature to identify and evaluate antenatal TB screening methods for pregnant women.

## METHODS

The protocol for this systematic review was registered with Prospero (CRD42024523017) on 21 March 2024. This study followed the Preferred Reporting Items for Systematic Reviews and Meta-analyses (PRISMA) reporting guidelines.^14^

### Search Methodology

Ovid MEDLINE, Embase + Embase Classic, Web of Science, and Cochrane Central Register of Controlled Trials (CENTRAL) were systematically searched by AM from database inception to 5 April 2024. Full search terms are described in the Supplementary Data. Searches of the electronic databases were supplemented by manual review of the reference lists of included articles for additional eligible studies.

### Primary and secondary outcomes

We included studies in which the primary outcome was antenatal TB screening. The population of interest was pregnant women, with the exposure and comparator being different methods of TB screening. Primary outcomes were methods and yield of active TB screening during the antenatal period. Active TB disease diagnosis was classified as either microbiologically confirmed TB or clinically diagnosed based on clinical symptoms and/or supportive investigations such as chest X-ray findings. Secondary outcomes include diagnostic tests, feasibility, compliance, cost-effectiveness, and pregnant women and healthcare workers perspectives on active TB screening during pregnancy.

### Study inclusion and exclusion criteria

Randomised control trials, cohort studies, case-control studies, cross-sectional studies, and descriptive studies were eligible for inclusion. For studies evaluating the symptoms screen, all symptom screening methods were included and were not restricted to the WHO-recommended symptom screen. Case reports, letters to the editor, commentaries, and conference abstracts were excluded, as were studies reporting TB disease in pregnancy incidentally found outside screening programs. In cases where multiple publications used the same data, the most recent study was included, providing additional information was not included in earlier publications. There were no restrictions on the study location, publication date or language.

### Screening and study selection

Results from each database were imported into the reference management software EndNote 20 (Clarivate, Philadelphia, PA, USA) and deduplicated. Covidence systematic review software was used for title and abstract, and full-text review screening. After removing duplicates, two reviewers (AM and GT) independently screened all titles and abstracts of retrieved studies and excluded studies according to the eligibility criteria. Full-text articles for the remaining studies were obtained and assessed for eligibility by two reviewers (AM, GT). If consensus could not be achieved, disagreements were resolved by a third reviewer (JD).

### Data extraction

Two reviewers (AM and GT) manually extracted data from eligible studies into a predefined Microsoft Excel data collection spreadsheet. Author(s), year of publication, country(ies) where the study was conducted, years of study recruitment, study design, sample size, and primary outcomes were recorded. TB incidence was obtained from national notification data for the year of the study; where studies were conducted over more than one calendar year, incidence from the mid-point year of the study was selected. Any discrepancies in data extraction were resolved following discussion with a third reviewer (JD).

### Risk of bias (quality assessment)

The quality of the included studies was assessed independently by two reviewers (AM and GT). Given the inclusion of diverse study designs, we assessed study quality using an eight-point checklist, adapted from the Newcastle-Ottawa Scale (NoS) for cohort and cross-sectional studies (Supplementary Data S2), and the Critical Appraisal Skills Program (CASP) for qualitative and mixed methods studies (S3). For the NoS, one point was assigned for each checklist item, with the overall study quality score calculated based on the sum of these points. We considered scores of 0 to 2 to be poor quality, 3 to 5 to be fair quality, and 6 to 8 to be good quality. For the CASP, we assessed confidence in qualitative and mixed methods studies by assessing study aims, methodology, design, recruitment, data collection, data analysis, reflexivity, ethical considerations, findings, and research contribution. After assessing each domain, we judged the risk of bias into four levels: no concerns, very minor concerns, minor concerns, and serious concerns.

### Evidence synthesis and statistical analysis

Meta-analysis was conducted on our primary outcomes to calculate yield, the number needed to screen (NNS), and positive predictive value (PPV). However, due to the high methodological heterogeneity, meta-analyses could not be performed on secondary outcomes (cost, feasibility, acceptability, and implementation); therefore, a narrative synthesis was conducted to summarise these findings.

The primary outcome measure was active TB case yield, defined as the number of pregnant women positive for active TB disease (microbiologically confirmed or clinically diagnosed) per 1,000 pregnant women screened. Random-effects meta-analysis was used to produce pooled estimates and 95% confidence intervals (CI) of yield per 1,000 women, stratified by screening method. A Freeman-Tukey double-arcsine transformation was applied to the proportions for variance stabilization, ^15^ and a random-effects model was applied to account for the high heterogeneity expected between studies. ^16^ Each study was weighted by its inverse variance, applying the Restricted Maximum Likelihood (REML) estimator. Between-study variance was estimated by τ^2^ and heterogeneity across studies was assessed by the conventional Chi-squared test for heterogeneity and the I^2^ statistic, which accounts for the number of studies included in the meta-analysis and provides a direct measure of unexplained variability. Forest plots were produced to demonstrate variation in the estimates and 95% CIs for yield in each included study. The number of pregnant women that would need to be screened via each method to detect one case of active TB was calculated as the reciprocal of the pooled estimates of the yield and corresponding 95% CIs. The PPV of each screening tool was calculated as the number of true positives (microbiologically confirmed or clinically diagnosed) divided by the sum of true positives and false positives (positive to screening tool, however, microbiologically negative or no clinical diagnosis). Forest plots of subgroup analyses for yield and PPV, stratified by HIV prevalence, and low versus high TB prevalence, were generated to visualise potential correlations between HIV status, TB prevalence, and the primary outcomes. All statistical analysis was performed using STATA version 18.0 (StataCorp, College Station, TX, USA). ^17^

This systematic review and meta-analysis utilized publicly available data from published studies and did not require ethical approval.

## RESULTS

### Search results and study characteristics

**Figure 1.**
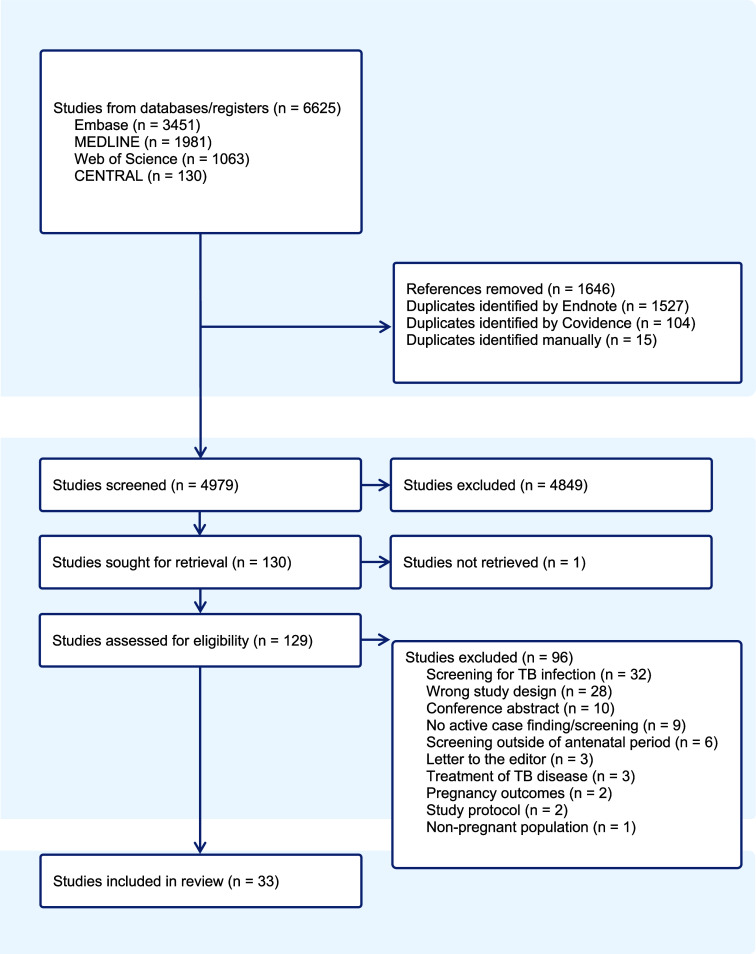
PRISMA flow diagram. Flowchart describing the inclusion of studies related to antenatal TB screening identified from the search strategy.

After the removal of duplicates, the initial search produced 4,979 results. After title and abstracts were screened, 129 full-text articles were reviewed, and 33 articles met the inclusion criteria (Figure 1). This includes 17 cross-sectional studies, 11 cohort studies, four mixed methods studies, and one qualitative study. Of these, 29 reported methods and yield of antenatal TB screening, one investigated cost, five examined feasibility, five addressed acceptability, and three reported on implementation. Ten studies reported from low TB incidence settings (<100 cases of TB per 100,000 individuals), whereas 23 reported from high TB incidence settings (≥100 cases of TB per 100,000 individuals). While our study methodology did not exclude extrapulmonary disease, only studies reporting pulmonary disease were identified. Characteristics of the included articles are summarised in Supplementary Data S4.

### Primary outcome and meta-analysis

#### Methods and yield of antenatal TB screening

Twenty-nine studies reported on antenatal TB screening methods and yield, identifying six screening approaches used during pregnancy (Supplementary Data S5). The most commonly employed methods were the symptom screen with 19 studies, and the tuberculin skin test (TST)/interferon gamma-release assay (IGRA) with 6 studies. The remaining methods, each reported by only one study, were excluded from formal analysis due to a lack of comparative data. Among the 19 studies that examined the symptom screen,^13,18–34^ sample sizes ranged from 63 to 113,078 pregnant women, with a combined total of 231,489 (Table 1). The number of reported TB cases identified by symptom screen ranged from 0 to 35. Among the 6 studies that examined the TST/IGRA,^35–40^ the number of pregnant women screened ranged from 176 to 7,638, with a combined total of 16,186 pregnant women. The number of reported TB cases identified by TST/IGRA ranged from 0 to 10. The pooled estimate for the yield of TB across all symptom screen studies was 7.26 (95% CI: 0.70, 19.25) per 1,000 pregnant women, compared to 5.12 (95% CI: 0.79, 12.39) for the TST/IGRA. This corresponds to 138 (95% CI: 52, 1,429) pregnant women needing to be screened with a symptom screen to detect one case of active TB. In contrast, the pooled estimate for TST/IGRA was significantly higher, with 1,667 (95% CI: 538, 1,000,000] pregnant women needing to be screened to identify a single case of active TB. The pooled PPV across all studies for a positive symptom screen to diagnose one case of active TB was 3.85% (95% CI: 1.23–7.57%), compared to <0.01% (95% CI: <0.01–0.05%) for the TST/IGRA (Table)

**Table.** Symptom screen vs. tuberculin skin test (TST) or interferon gamma-release assay (IGRA). Pooled summary statistics and the estimates and 95% confidence intervals (CI) of active TB case yield, number needed to screen (NNS) to identify a single active TB case, and positive predictive value (PPV).

Screening method	Symptom screen	TST/IGRA
Total screened, n	231,489	16,186
Positive to screening test, n (%)	15,639 (6.8)	12,472 (77.1)
Positive to active TB, among those who screened positive, n (%)	176 (1.1)	22 (0.2)
Yield per 1,000, n [95% CI]	7.26 [0.70, 19.25]	5.12 [0.79, 12.39]
NNS to identify one case, n [95% CI]	138 [52, 1,429]	1,667 [538, 1,000,000]
PPV, % [95% CI]	3.85 [1.23, 7.57]	<0.01 [<0.01, 0.05]

n = number; CI = confidence interval; NNS = number needed to screen; PPV = positive predictive value.

**Figure 2.**
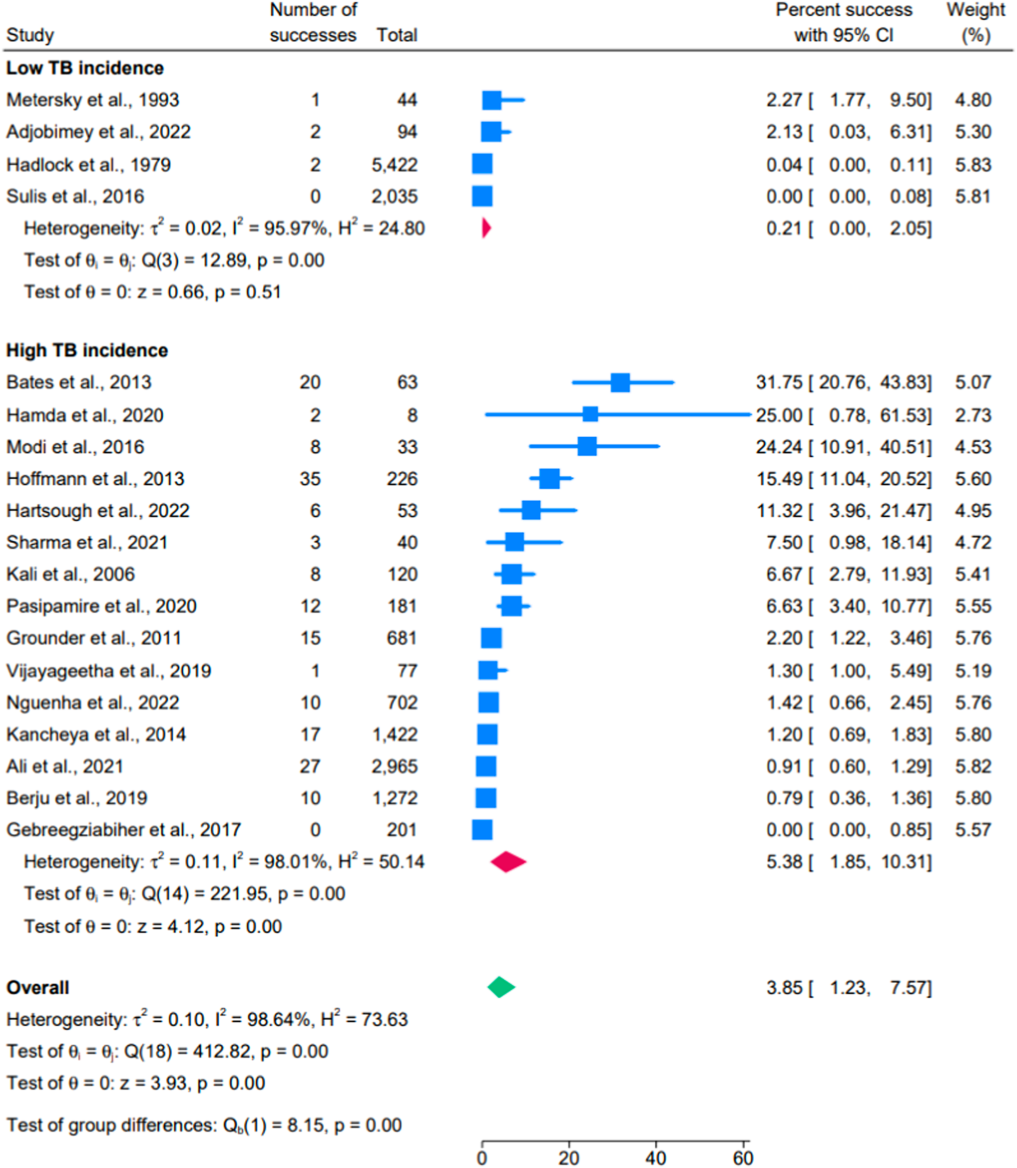
Forest plot of positive predictive value of antenatal TB symptom screening. Forest plot of the Positive Predictive Value (PPV) of antenatal TB symptom screening, stratified by low and high TB prevalence in the country of study. The number of successes refers to individuals diagnosed with TB disease, and the total represents those who tested positive on the symptom screen. The red diamonds indicate the estimated group-specific pooled PPV, while the green diamond represents the estimated overall PPV. The blue boxes show the individual study estimates, and the blue lines denote the 95% confidence intervals (CI).

We conducted subgroup analyses on the PPV of symptom screen based on TB incidence in the country of study (Figure 2). The pooled PPV of the symptom screen within high TB incidence settings was 5.83% (95% CI: 1.85–10.31%) compared to 0.21% (95% CI: <0.00–2.05%) in low TB incidence settings. All studies using TST/IGRA were conducted in low TB incidence settings, so no sub-group analysis based on TB incidence was performed. Additionally, the forest plot stratifying HIV status and the yield of active TB cases revealed no visually discernible correlation (Supplementary Data S6).

The meta-analyses revealed substantial statistical heterogeneity among studies, with the I^2^ ranging between 9.53%-99.56% (Supplementary Data S6).

### Secondary outcomes and narrative synthesis

#### Cost

A cross-sectional study from Benin included a cost assessment of routine antenatal TB screening.^18^ Of the 4,070 pregnant women screened, 94 (2.3%) were symptomatic, of which 2 (2.1%) had microbiologically confirmed TB.^18^ The authors reported that, from a health systems perspective, the average cost to screen one pregnant woman for TB using a symptom-based tool was US$1.12, whereas the cost per microbiologically confirmed TB case was US$2,271.^18^

#### Feasibility

Five studies conducted in high TB incidence areas evaluated the feasibility of integrating antenatal TB screening into existing reproductive, maternal, newborn and child health (RMNCH) care settings.^13,18,26,33,41^ Feasibility was defined as how easily the antenatal screening program could be implemented based on its operationalisation and available support at the site.^13,18,26,33,41^ All five studies concluded that integrating routine antenatal TB screening is feasible. A mixed-methods study from Eswatini examined the integration of TB/HIV services within RMNCH settings and found it both feasible and effective in ensuring high TB screening coverage among women of reproductive age.^26^ This aligns with the findings of a cross-sectional study in Zambia, which confirmed the feasibility of TB symptom screening among women attending RMNCH services.^13^

#### Acceptability

Five studies in high-TB incidence settings assessed the acceptability of routine TB screening integrated into existing maternal care among pregnant women^13,18,26,41,42^ and found that most women and healthcare workers were receptive to antenatal TB screening. One cross-sectional study of 2,044 pregnant women in Mozambique found that 1,980 (96.9%) agreed to symptom screening. ^41^ Similarly, a study in Zambia found a 100% acceptance rate, with all 5,033 pregnant women undergoing symptom screening.^13^ A pilot study in Eswatini^26^ indicated that local healthcare workers deemed routine TB screening for pregnant women acceptable. Qualitative analysis indicated that 96% of healthcare workers supported the integration of TB screening, 47% found it easy to integrate into existing maternity care services, and 37% considered the ongoing delivery of TB screening highly acceptable.^26^ This was supported by a study in Benin where routine antenatal TB screening was deemed acceptable by maternity service providers.^18^

#### Implementation

Three studies evaluated the implementation of antenatal TB screening into existing maternal services and identified barriers to successful integration.^43–45^ Two studies conducted in South Africa reported inadequate implementation of antenatal TB screening.^43, 45^ Despite integration into already existing HIV services, antenatal TB screening efforts were suboptimal.^43,45^ Peters et al. reported that women who underwent HIV testing were 30% less likely to be screened for TB.^43^ The authors conclude that the unexpected reluctance to screen HIV-positive women for TB may stem from a combination of ethical concerns such as informed consent and confidentiality, psychological factors including fear of additional stigma and increased patient burden, and practical considerations like time constraints and complexity of managing multiple conditions.^43^ Both studies report that the barriers identified included gaps in training, poor coordination between healthcare systems, and insufficient resources.^43^ One prospective cohort study from Lesotho found that TB antenatal screening was easily integrated into RMNCH, however, further resources would be required for adequate follow-up of pregnant women positive for TB.^44^

## DISCUSSION

Of the approaches to antenatal TB disease screening identified in the literature, symptom screening demonstrates the highest yield and efficiency. We found that symptom screening is low cost, feasible and acceptable to both healthcare workers and pregnant women, and currently is the most widely preferred antenatal approach to active TB screening. Despite these benefits, the PPV of this method is low, highlighting key opportunities for the development of improved screening methodologies in future.

The standardised WHO four-point symptom screen was initially developed to help exclude TB in adults living with HIV.^12^ In non-pregnant populations, the PPV of this symptom screen varies significantly based on the demographics of the individuals being screened and the local incidence of TB.^12,46,47^ Sub-group analysis within our study demonstrates a higher PPV for the symptom screen in high TB incidence settings (5.38%, 95% CI: 1.85–10.31%) compared to low incidence settings (0.21%, 95% CI: <0.01–2.05%) see Figure 2, although regardless of disease endemicity, the PPV for the symptom screen is poor during pregnancy. Studies have shown that the signs and symptoms of TB in pregnant women can differ from those in non-pregnant individuals.^48,49^ This variation and poor performance of the existing tool underscore the need for a specific TB screening tool tailored to pregnant populations. Such a tool could enhance the accuracy of diagnosis by considering pregnancy-related symptom presentations, ultimately leading to timely identification and treatment of active TB. Future research should focus on this and identify barriers to TB screening implementation in antenatal care.

Alternative approaches to identifying TB, including blood- or urine-based diagnostic tests, may also be useful to pursue. Although our study demonstrated the poor performance of TST/IGRA as lone screening tools for active TB in pregnancy, they may be considered as part of screening programs for identifying TB infection and prevention through targeted treatment programs in appropriate contexts.^9^ Evaluation of other emerging approaches in pregnancy, such as the use of urine LAM or circulating microRNA, should also be considered in antenatal contexts to ensure that pregnant women are able to rapidly benefit from diagnostic advances.^50,51^

Key limitations of our study include the inability to calculate sensitivity, specificity and negative predictive value due to insufficient data, highlighting the need for improved bidirectional data collection between TB and maternity care settings. Also, limited data on 4 of the 6 identified screening methods restricted our comparative analysis to symptom screening and IGRA/TST approaches, potentially overlooking the other 4 strategies due to insufficient evidence. Additionally, the distribution of study settings may limit the generalizability of findings. Furthermore, the heterogeneity of study designs, including some that selectively screened individuals at higher risk rather than conducting unbiased screening of the general pregnant population, may have biased the reported yield of TB cases.

## CONCLUSION

In pregnant women, the symptom screen demonstrates the highest yield, with an estimated 7.26 (95% CI: 0.70, 19.25) cases detected per 1,000 pregnant women, and the lowest number needed to screen to identify one case of active TB at 138 (95% CI: 52, 1,429) women. Due to its low cost, high feasibility, and widespread acceptability, the symptom screen is the preferred method for antenatal screening of TB disease. However, the low PPV of 3.85% (95% CI: 1.23%, 7.57%) underscores the need for a TB screening tool with greater sensitivity specifically tailored to pregnant populations.

## Supplementary Material


