# Supplementary material for: Antenatal screening for TB disease: a systematic review and meta-analysis
Source: IJTLD Open. 2025 Jun 13;2(6):366–73. doi: 10.5588/ijtldopen.25.0105 (PMC12168728; doi:10.5588/ijtldopen.25.0105)
Supplement: Supplementary file 1 [file ijtldopen25-0105_supplementarydata1.pdf]

Appendix S1. Search Strategy

Ovid MEDLINE

**Search Number**<br>Results	Search Terms
**1**<br>1,429,022	pregnancy/ OR puerperium/ OR parturition/ OR antepartum period/ OR obstetrics/
**2**<br>1,162,295	pregnan*.mp. [mp=title, abstract, original title, name of substance word, subject heading word, floating sub-heading word, keyword heading word, organism supplementary concept word, protocol supplementary concept word, rare disease supplementary concept word, unique identifier, synonyms]
**3**<br>27,046	puerper*.mp. [mp=title, abstract, original title, name of substance word, subject heading word, floating sub-heading word, keyword heading word, organism supplementary concept word, protocol supplementary concept word, rare disease supplementary concept word, unique identifier, synonyms]
**4**<br>38,339	parturi*.mp. [mp=title, abstract, original title, name of substance word, subject heading word, floating sub-heading word, keyword heading word, organism supplementary concept word, protocol supplementary concept word, rare disease supplementary concept word, unique identifier, synonyms]
**5**<br>6,965	antepartum.mp. [mp=title, abstract, original title, name of substance word, subject heading word, floating sub-heading word, keyword heading word, organism supplementary concept word, protocol supplementary concept word, rare disease supplementary concept word, unique identifier, synonyms]
**6**<br>212,888	obstetric*.mp. [mp=title, abstract, original title, name of substance word, subject heading word, floating sub-heading word, keyword heading word, organism supplementary concept word, protocol supplementary concept word, rare disease supplementary concept word, unique identifier, synonyms]
**7**<br>303,040	mycobacterium tuberculosis/ OR tuberculosis/ OR active tuberculosis/ OR tuberculosis disease
**8**<br>77,384	mycobacterium tuberculosis.mp. [mp=title, abstract, original title, name of substance word, subject heading word, floating sub-heading word, keyword heading word, organism supplementary concept word, protocol supplementary concept word, rare disease supplementary concept word, unique identifier, synonyms]
**9**<br>283,538	tuberculosis.mp. [mp=title, abstract, original title, name of substance word, subject heading word, floating sub-heading word, keyword heading word, organism supplementary concept word, protocol supplementary concept word, rare disease supplementary concept word, unique identifier, synonyms]
**10**<br>5,051	active tuberculosis.mp. [mp=title, abstract, original title, name of substance word, subject heading word, floating sub-heading word, keyword heading word, organism supplementary concept word, protocol supplementary concept word, rare disease supplementary concept word, unique identifier, synonyms]
**11**<br>1,300	tuberculosis disease.mp. [mp=title, abstract, original title, name of substance word, subject heading word, floating sub-heading word, keyword heading word, organism supplementary concept word, protocol supplementary concept word, rare disease supplementary concept word, unique identifier, synonyms]
**12**<br>4,887,383	screening/ OR diagnosis/

**13**<br>794,911	screening.mp. [mp=title, abstract, original title, name of substance word, subject heading word, floating sub-heading word, keyword heading word, organism supplementary concept word, protocol supplementary concept word, rare disease supplementary concept word, unique identifier, synonyms]
**14**<br>4,300,130	diagnosis.mp. [mp=title, abstract, original title, name of substance word, subject heading word, floating sub-heading word, keyword heading word, organism supplementary concept word, protocol supplementary concept word, rare disease supplementary concept word, unique identifier, synonyms]
**15**<br>1,491,105	#1 OR #2 OR #3 OR #4 OR #5 OR #6
**16**<br>303,040	#7 OR #8 OR #9 OR #10 OR #11
**17**<br>4,887,383	#12 OR #13 OR #14
**18**<br>1,981	#15 AND #16 AND #17
Total	1,981

Embase + Embase Classic

**Search Number**<br>Results	Search Terms
**1**<br>1,643,024	pregnancy/ OR puerperium/ OR parturition/ OR antepartum period/ OR obstetrics/
**2**<br>1,299,747	pregnan*.mp. [mp=title, abstract, original title, name of substance word, subject heading word, floating sub-heading word, keyword heading word, organism supplementary concept word, protocol supplementary concept word, rare disease supplementary concept word, unique identifier, synonyms]
**3**<br>78,860	puerper*.mp. [mp=title, abstract, original title, name of substance word, subject heading word, floating sub-heading word, keyword heading word, organism supplementary concept word, protocol supplementary concept word, rare disease supplementary concept word, unique identifier, synonyms]
**4**<br>34,202	parturi*.mp. [mp=title, abstract, original title, name of substance word, subject heading word, floating sub-heading word, keyword heading word, organism supplementary concept word, protocol supplementary concept word, rare disease supplementary concept word, unique identifier, synonyms]
**5**<br>11,661	antepartum.mp. [mp=title, abstract, original title, name of substance word, subject heading word, floating sub-heading word, keyword heading word, organism supplementary concept word, protocol supplementary concept word, rare disease supplementary concept word, unique identifier, synonyms]
**6**<br>244,545	obstetric*.mp. [mp=title, abstract, original title, name of substance word, subject heading word, floating sub-heading word, keyword heading word, organism supplementary concept word, protocol supplementary concept word, rare disease supplementary concept word, unique identifier, synonyms]
**7**<br>383,087	mycobacterium tuberculosis/ OR tuberculosis/ OR active tuberculosis/ OR tuberculosis disease
**8**<br>109,871	mycobacterium tuberculosis.mp. [mp=title, abstract, original title, name of substance word, subject heading word, floating sub-heading word, keyword heading word, organism supplementary concept word, protocol

	supplementary concept word, rare disease supplementary concept word, unique identifier, synonyms]
**9**<br>364,410	tuberculosis.mp. [mp=title, abstract, original title, name of substance word, subject heading word, floating sub-heading word, keyword heading word, organism supplementary concept word, protocol supplementary concept word, rare disease supplementary concept word, unique identifier, synonyms]
**10**<br>7,450	active tuberculosis.mp. [mp=title, abstract, original title, name of substance word, subject heading word, floating sub-heading word, keyword heading word, organism supplementary concept word, protocol supplementary concept word, rare disease supplementary concept word, unique identifier, synonyms]
**11**<br>1,703	tuberculosis disease.mp. [mp=title, abstract, original title, name of substance word, subject heading word, floating sub-heading word, keyword heading word, organism supplementary concept word, protocol supplementary concept word, rare disease supplementary concept word, unique identifier, synonyms]
**12**<br>7,933,948	screening/ OR diagnosis/
**13**<br>1,409,974	screening.mp. [mp=title, abstract, original title, name of substance word, subject heading word, floating sub-heading word, keyword heading word, organism supplementary concept word, protocol supplementary concept word, rare disease supplementary concept word, unique identifier, synonyms]
**14**<br>6,925,381	diagnosis.mp. [mp=title, abstract, original title, name of substance word, subject heading word, floating sub-heading word, keyword heading word, organism supplementary concept word, protocol supplementary concept word, rare disease supplementary concept word, unique identifier, synonyms]
**15**<br>1,781,280	#1 OR #2 OR #3 OR #4 OR #5 OR #6
**16**<br>383,087	#7 OR #8 OR #9 OR #10 OR #11
**17**<br>7,933,948	#12 OR #13 OR #14
**18**<br>3,451	#15 AND #16 AND #17
Total	3,451

Web of Science

**Search Number**<br>Results	Search Terms
**1**<br>966,873	ALL=(pregnan*) OR ALL=(puerper*) OR ALL=(parturi*) OR ALL=(antepartum) OR ALL=(obstetric*)
**2**<br>249,940	ALL=(mycobacterium tuberculosis) OR ALL=(tuberculosis) OR ALL=(active tuberculosis) OR ALL=(tuberculosis disease)
**3**<br>3,589,403	ALL=(screening) OR ALL=(diagnosis)
**4**<br>167	#1 AND #2 AND #3
Total	1,063

Cochrane Central Register of Controlled Trials (CENTRAL)

**Search Number**<br>Results	Search Terms
**1**<br>96,295	(pregnan*):ti,ab,kw OR (puerper*):ti,ab,kw OR (parturi*):ti,ab,kw OR (antepartum):ti,ab,kw OR (obstetric*):ti,ab,kw
**2**<br>1,115	("Mycobacterium tuberculosis"):ti,ab,kw
**3**<br>508	MeSH descriptor: [Mycobacterium tuberculosis] explode all trees
**4**<br>332,961	("screening"):ti,ab,kw OR (diagnosis):ti,ab,kw
**5**<br>5	MeSH descriptor: [Diagnostic Screening Programs] explode all trees
**6**<br>1,115	#2 OR #3
**7**<br>332,961	#4 OR #5
**8**<br>130	#1 AND #6 AND #7
Total	130

Appendix S2. Newcastle-Ottawa Scale (Modified Version)

Conflicts of Interest

Q1. No evidence of conflicts of interest by one or more authors

1. Yes
0. No

Selection

Q2. Representative of the population of interest.

1. Yes

0. No

Q3. Cohort characteristics are well described.

1. Yes

0. No

Q4. Ascertainment of the exposure/comparator is well described.

1. Yes

0. No

Comparability

Q5. If heterogeneous groups were included in the study cohort, results were disaggregated by these characteristics.

1. Yes

0. No

Outcome

Q6. Definition of outcome is well described.

1. Yes

0. No

Q7. Was the duration of the study appropriate to assess the outcome of interest?

1. Yes

0. No

Q8. Adequacy of follow-up of cohort - follow-up of ≥80% of participants

1. Yes

0. No

Overall study quality score

0-2 Poor

3-5 Fair

6-8 Good

Study author, year	Q1	Q2	Q3	Q4	Q5	Q6	Q7	Q8	Overall study quality score
Ali et al., 2021	1	1	1	1	1	1	1	1	8
Bates et al., 2013	0	1	1	1	1	0	1	1	6
Batshake et al., 2023	1	1	1	1	1	1	1	1	8
Berju et al., 2019	1	1	1	1	1	1	0	1	7
Bullarbo et al., 2018	1	1	1	1	1	1	1	1	8
Froberg et al. 2020	1	1	1	1	1	1	1	1	8
Gebreegziabiher et al., 2017	1	1	1	1	1	1	1	1	8
Grounder et al., 2011	1	1	1	1	1	1	1	1	8
Hadlock et al., 1979	0	1	0	1	0	0	1	1	4
Hamda et al., 2020	1	0	1	0	1	1	1	1	6
Hoffmann et al., 2013	1	1	1	1	1	1	1	1	8
Kali et al., 2006	0	1	0	1	0	1	1	1	5
Kancheya et al., 2014	1	1	1	0	1	0	1	1	6
Kosgei et al., 2011	0	1	0	1	1	0	1	1	5
LaCourse et al., 2016	1	1	1	1	1	1	1	1	8
Metersky et al., 1993	0	1	0	1	0	0	1	1	4
Modi et al., 2016	1	1	1	1	1	1	1	1	8
Montgomery et al., 1968	0	1	0	1	0	0	1	1	4
Nguenha et al., 2022	0	1	1	1	1	1	1	1	7
Pasipamire et al., 2020	1	1	1	1	1	1	1	1	8
Peters et al., 2015	1	0	0	1	1	1	1	1	6
Plauche et al., 1983	0	0	1	0	0	1	1	1	4
Schechner et al., 2015	0	1	1	1	0	1	1	1	6
Schulte et al., 2002	0	1	0	1	0	1	1	1	5
Schwartz et al., 2009	0	1	0	1	0	0	1	1	4
Sharma et al., 2021	0	1	1	1	1	1	1	1	7
Sulis et al., 2016	0	1	0	1	0	1	1	1	5
Tiam et al., 2014	0	1	1	1	1	1	1	1	7

Appendix S3. CASP assessments of qualitative and mixed methods studies

Lead author and year	Was there a clear statement of the aims of the research?	Is a qualitative methodology appropriate?	Was the research design appropriate	Was the recruitment strategy appropriate to the aims of the research?	Was the data collected in a way that addressed the research issue?	Has the relationship between researcher and participants been adequately considered?	Have ethical issues been taken into consideration?	Was the data analysis sufficiently rigorous?	Is there a clear statement of findings?	How valuable is the research?	Overall concerns about methodological limitations
**Adjobimey et al., 2022**	Yes	Yes	Yes	Yes	Yes	Can't tell	Yes	Yes	Yes	Valuable	No or very minor concerns
**Hartsough et al., 2022**	Yes	Yes	Yes	Yes	Yes	No	Yes	Yes	Yes	Somewhat valuable	Minor concerns
**Sangala et al., 2006**	Yes	Yes	Yes	Yes	Yes	No	No	Partial	Yes	Valuable	Minor concerns
**Uwimana et al., 2013**	Yes	Yes	Yes	Yes	Yes	No	Yes	Yes	Yes	Somewhat valuable	Minor concerns
**Vijayageetha et al., 2019**	Yes	Yes	Yes	Yes	Yes	Yes	Yes	Partial	Yes	Valuable	No or very minor concerns

Appendix S4. Characteristics of the included studies

No.	Author, year	Country/ies	Years of study recruitment	Study Design	Sample size n	Included outcomes	Study quality
1	Adjobimey et al., 2022	Benin	2017-2018	Mixed-methods	4,070	Methods, yield, cost, feasibility, and acceptability	No or very minor concerns
2	Ali et al., 2021	Pakistan	2017	Cross-sectional	2,896	Methods, yield	Good
3	Bates et al., 2013	Zambia	n/s	Prospective cohort	94	Methods, yield	Good
4	Batshake et al., 2023	Sweden	204-2018	Retrospective cohort	7,638	Methods, yield	Good
5	Berju et al., 2019	Ethiopia	2015-2016	Cross-sectional	1,272	Methods, yield	Good
6	Bullarbo et al., 2018	Sweden	2008-2012	Retrospective cohort	902	Methods, yield	Good
7	Froberg et al. 2020	Sweden	2016-2017	Retrospective cohort	4,178	Methods, yield	Good
8	Gebreegziabiher et al., 2017	Ethiopia	2014-2015	Cross-sectional	9,600	Methods, yield	Good
9	Grounder et al., 2011	South Africa	2008-2009	Cross-sectional	3,963	Methods, yield	Good
10	Hadlock et al., 1979	USA	1976-1977	Retrospective cohort	5,422	Methods, yield	Fair
11	Hamda et al., 2020	Botswana	2017-2018	Cross-sectional	407	Methods, yield	Good
12	Hartsough et al., 2022	Eswatini	2017	Mixed-methods	3,361	Methods, yield, feasibility, and acceptability	Minor concerns
13	Hoffmann et al., 2013	South Africa	2010-2011	Cross-sectional	1,415	Methods, yield	Good
14	Kali et al., 2006	South Africa	2003	Cross-sectional	370	Methods, yield	Fair
15	Kancheya et al., 2014	Zambia	2011-2012	Cross-sectional	5,033	Methods, yield, feasibility, and acceptability	Good
16	Kosgei et al., 2011	Kenya	2009-2010	Cross-sectional	187	Methods, yield	Fair
17	LaCourse et al., 2016	Kenya	2013-2014	Cross-sectional	306	Methods, yield	Good
18	Metersky et al., 1993	USA	1990-1991	Prospective cohort	1,533	Methods, yield	Fair
19	Modi et al., 2016	Kenya	2011-2012	Prospective cohort	738	Methods, yield	Good
20	Montgomery et al., 1968	USA	1960-1966	Cross-sectional	142	Methods, yield	Fair
21	Nguenha et al., 2022	Mozambique	2016-2018	Cross-sectional	1,980	Methods, yield, feasibility, and acceptability	Good
22	Pasipamire et al., 2020	Eswatini	2015	Cross-sectional	990	Methods, yield	Good
23	Peters et al., 2015	South Africa	2011-2012	Cross-sectional	308	Implementation	Good
24	Plauche et al., 1983	USA	1981-1982	Cross-sectional	1,935	Methods, yield	Fair
25	Sangala et al., 2006	Malawi	n/s	Qualitative	54	Acceptability	Minor concerns
26	Schechner et al., 2015	Israel	2012-2013	Retrospective cohort	431	Methods, yield	Good
27	Schulte et al., 2002	USA	1995-1996	Cross-sectional	207	Methods, yield	Fair
28	Schwartz et al., 2009	USA	2001-2006	Retrospective cohort	4,049	Methods, yield	Fair
29	Sharma et al., 2021	India	2020	Prospective cohort	1,306	Method, yield	Good

Appendix S4. Characteristics of the included studies

30	Sulis et al., 2016	Burkina Faso	2014-2015	Cross-sectional	2,000	Methods, yield, and feasibility	Fair
31	Tiam et al., 2014	Lesotho	2011	Prospective cohort	800	Implementation	Good
32	Uwimana et al., 2013	South Africa	2008	Mixed-methods	150	Implementation	Minor concerns
33	Vijayageetha et al., 2019	India	2018	Mixed-methods	4,203	Methods, yield, and implementation	No or very minor concerns

Legend: n/s = not stated

Appendix S5. Methods and yield of antenatal TB screening

No.	Author, year	Study country	Study period y	Approx. active TB incidence per 100,000/annum*	Sample size n	HIV n (%)	Diagnostic method	Pregnant women screened n	Positive to symptom screen n (%)	Positive to TST/IGRA n (%)	Positive to CXR n (%)	Positive to GeneXpert/Culture n (%)	Active TB cases detected n (%)
1	Adjobimey et al., 2022	Benin	2017-2018	58	4,070	n/s	Symptom screen	4,070	94 (20.3)			2 (2.1)	2 (2.1)
2	Ali et al., 2021	Pakistan	2017	226	113,078	905 (0.8)	Symptom screen	113,078	2,965 (2.6)			27 (0.9)	27 (0.9)
3	Bates et al., 2013	Zambia	n/s	388	63	46 (73.0)	Symptom screen	63	63 (100.0)			20 (31.7)	20 (31.7)
4	Batshake et al., 2023	Sweden	2014-2018	8	7,638	44 (0.6)	TST/IGRA	7,638		7,638 (100.0)		10 (0.1)	10 (0.1)
5	Berju et al., 2019	Ethiopia	2015-2016	192	1,272	112 (8.8)	Symptom screen	1,272	1,272 (100.0)			10 (0.8)	10 (0.8)
6	Bullarbo et al., 2018	Sweden	2008-2012	6	902	n/s	TST/IGRA	902		327 (36.3)		1 (0.3)	1 (0.3)
7	Froberg et al. 2020	Sweden	2016-2017	8	4,178	39 (0.9)	TST/IGRA	4,178		4,178 (100.0)		9 (0.2)	9 (0.2)
8	Gebreegziabiher et al., 2017	Ethiopia	2014-2015	207	9,600	604 (6.3)	Symptom screen	9,600	201 (2.1)			0 (0.0)	0 (0.0)
9	Grounder et al., 2011	South Africa	2008-2009	1,270	3,963	1,454 (36.7)	Symptom screen	3,963	681 (0.2)			15 (2.2)	15 (2.2)
10	Hadlock et al., 1979	USA	1976-1977	27	5,422	n/s	Symptom screen	5,422	5,422 (100.0)		2 (0.04)		2 (0.04)
11	Hamda et al., 2020	Botswana	2017-2018	300	407	69 (17.0)	Symptom Screen	407	8 (2.0)			2 (25.0)	2 (25.0)
12	Hartsough et al., 2022	Eswatini	2017	502	3,361	1,945 (3.0)	Symptom screen	3,361	53 (1.6)			6 (11.3)	6 (11.3)
13	Hoffmann et al., 2013	South Africa	2010-2011	1,230	1,415	1,415 (100.0)	Symptom screen	1,415	226 (16.0)			35 (15.5)	35 (15.5)
14	Kali et al., 2006	South Africa	2003	1,070	370	370 (100.0)	Symptom screen	370	120 (32.4)			8 (6.6)	8 (6.6)
15	Kancheya et al., 2014	Zambia	2011-2012	475	5,033	855 (17)	Symptom screen	5,033	1,422 (28.3)			17 (1.2)	17 (1.2)
16	Kosgei et al., 2011	Kenya	2009-2010	566	187	187 (100)	Symptom screen + Chest x-ray	187	38 (20.3)		10 (26.3)		10 (26.3)
17	LaCourse et al., 2016	Kenya	2013-2014	443	306	306 (100)	Symptom screen + TST/IGRA	228	56 (24.6)	18 (32.1)		7 (38.9)	7 (38.9)
18	Metersky et al., 1993	USA	1990-1991	48	1,533	n/s	Symptom screen	1,533	44 (2.9)		12 (27.3)		1 (8.3)
19	Modi et al., 2016	Kenya	2011-2012	506	738	24 (3.3))	Symptom screen	134	33 (24.6)			8 (24.2)	8 (24.2)
20	Montgomery et al., 1968	USA	1960-1966	60	1,357	n/s	TST/IGRA	1,357		199 (14.7)	0 (0.0)		0 (0.0)
21	Nguenha et al., 2022	Mozambique	2016-2018	361	1,980	613 (31.0)	Symptom screen	1,980	702 (35.5)			10 (1.4)	10 (1.4)
22	Pasipamire et al., 2020	Eswatini	2015	648	990	254 (25.7)	Symptom screen	990	181 (18.3)			12 (6.6)	12 (6.6)
24	Plauche et al., 1983	USA	1981-1982	22	1,935	n/s	TST/IGRA	1,935		85 (4.4)	0 (0.0)		0 (0.0)
26	Schechner et al., 2015	Isael	2012-2013	8	431	n/s	Chest x-ray	431			11 (2.6)	3 (27.3)	3 (27.3)
27	Schulte et al., 2002	USA	1995-1996	32	207	207 (100)	TST/IGRA	176		45 (25.6)		2 (4.4)	2 (4.4)
28	Schwartz et al., 2009	USA	2001-2006	6	4,049	n/s	TST + chest x-ray	4,049		1,935 (47.8)	1 (0.05)	0 (0.0)	0 (0.0)
29	Sharma et al., 2021	India	2020	197	1,306	n/s	Symptom Screen	1,306	40 (3.1)			3 (7.5)	3 (7.5)
30	Sulis et al., 2016	Burkina Faso	2014-2015	54	73,289	n/s	Symptom screen	73,289	2,035 (2.8)			0 (0.0)	0 (0.0)

Appendix S5. Methods and yield of antenatal TB screening

33	Vijayageetha et al., 2019	India	2018	208	4,203	2 (0.05)	Symptom screen	4,203	77 (1.8)			1 (1.3)	1 (1.3)

Legend: n/s = not stated

Appendix S6. Additional figures

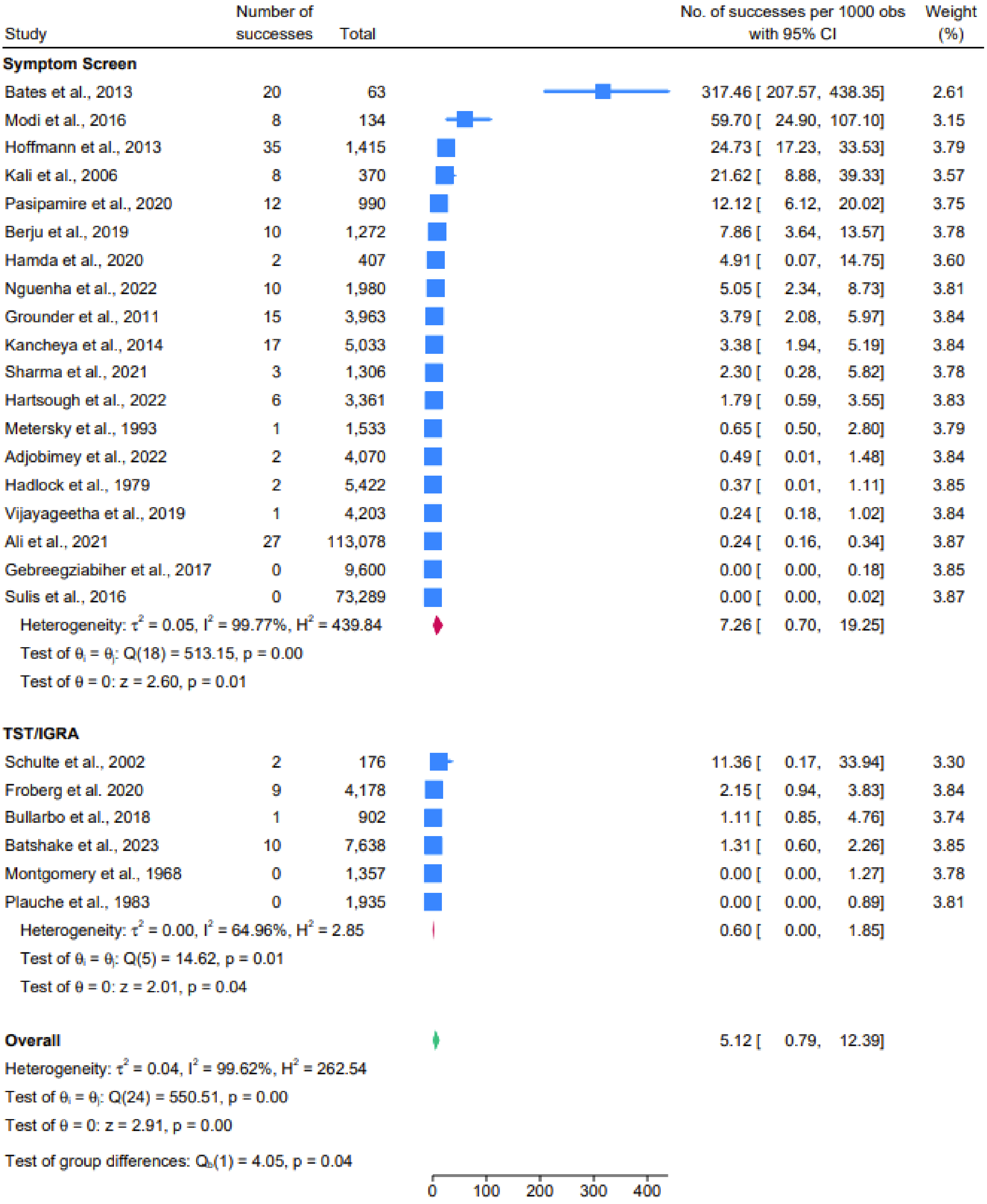

Supplementary Figure 1. Forest plot of active TB case yield per 1,000 pregnant women screened for TB antenatally, stratified by test methodology (symptom screen and Tuberculin Skin Test (TST)/Interferon Gamma-Release Assay (IGRA)). The number of successes refers to individuals screened as positive, and the total represents the total number of women screened. The red diamonds

indicate the estimated group-specific pooled yield, while the green diamond represents the estimated overall yield. The blue boxes show the individual study estimates, and the blue lines denote the 95% confidence intervals (CI).

Appendix S6. Additional figures

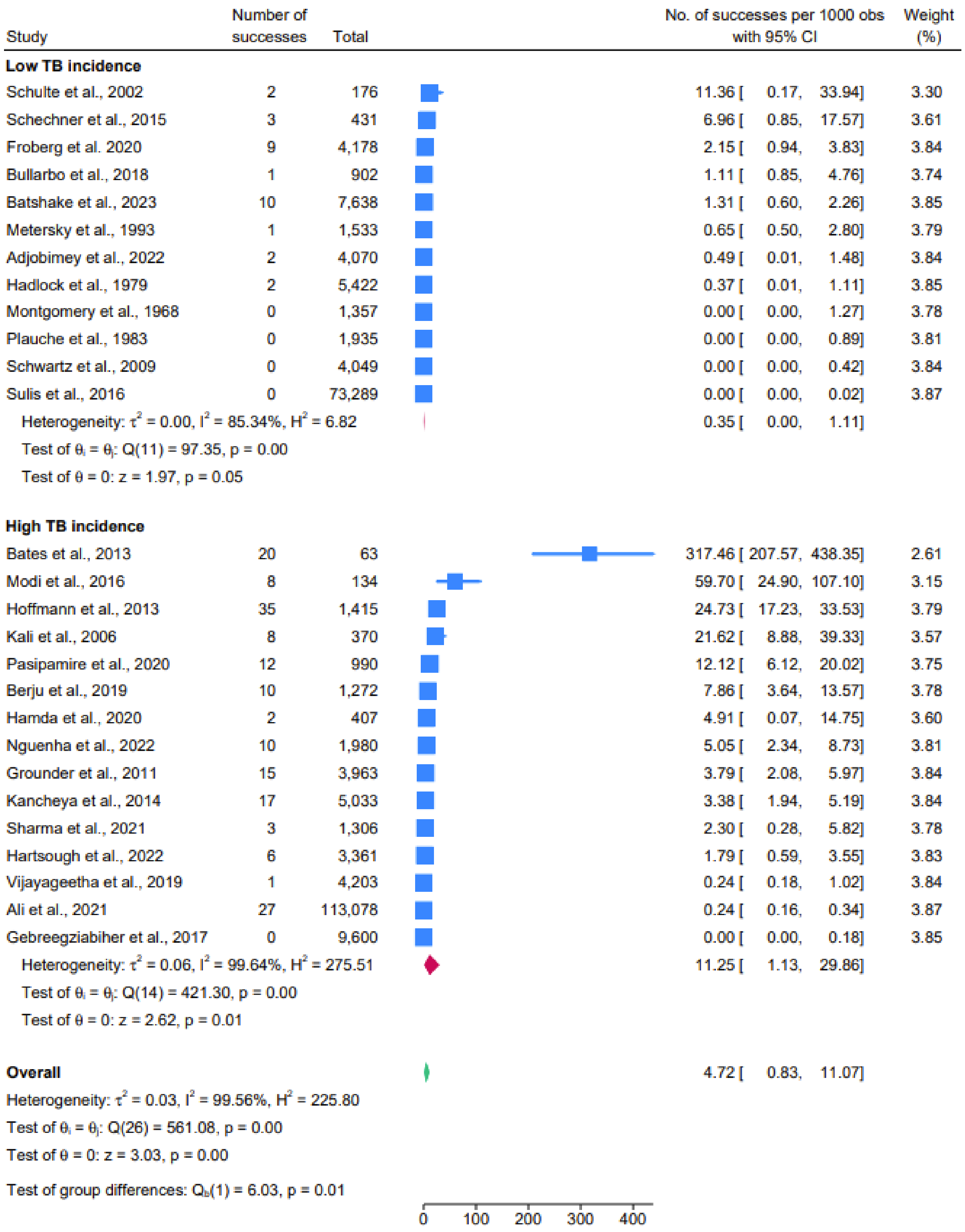

Supplementary Figure 2. Forest plot of the active TB case yield per 1,000 pregnant women screened for TB antenatally, stratified by low and high TB incidence in the country/countries of study. The number of successes refers to individuals screened as positive, and the total represents the total number of women screened. The red diamonds indicate the estimated group-specific pooled yield,

while the green diamond represents the estimated overall yield. The blue boxes show the individual study estimates, and the blue lines denote the 95% confidence intervals (CI).

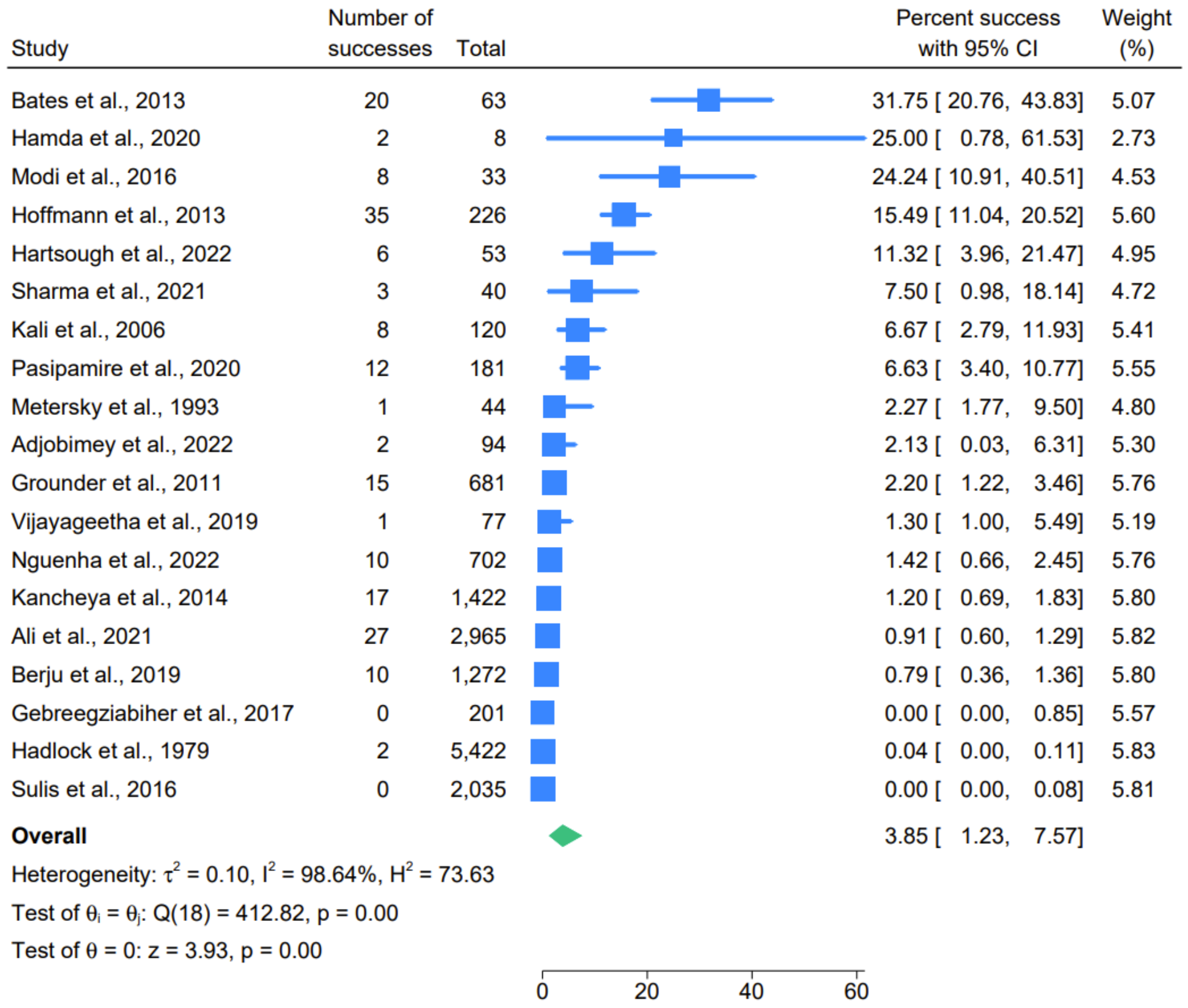

Supplementary Figure 3. Forest plot of the positive predictive value (PPV) of antenatal TB symptom screening. The number of successes refers to individuals diagnosed with TB disease, and the total represents those who tested positive in the symptom screen. The green diamond represents the estimated overall PPV. The blue boxes show the individual study estimates and the blue lines denote the 95% confidence intervals (CI).

Appendix S6. Additional figures

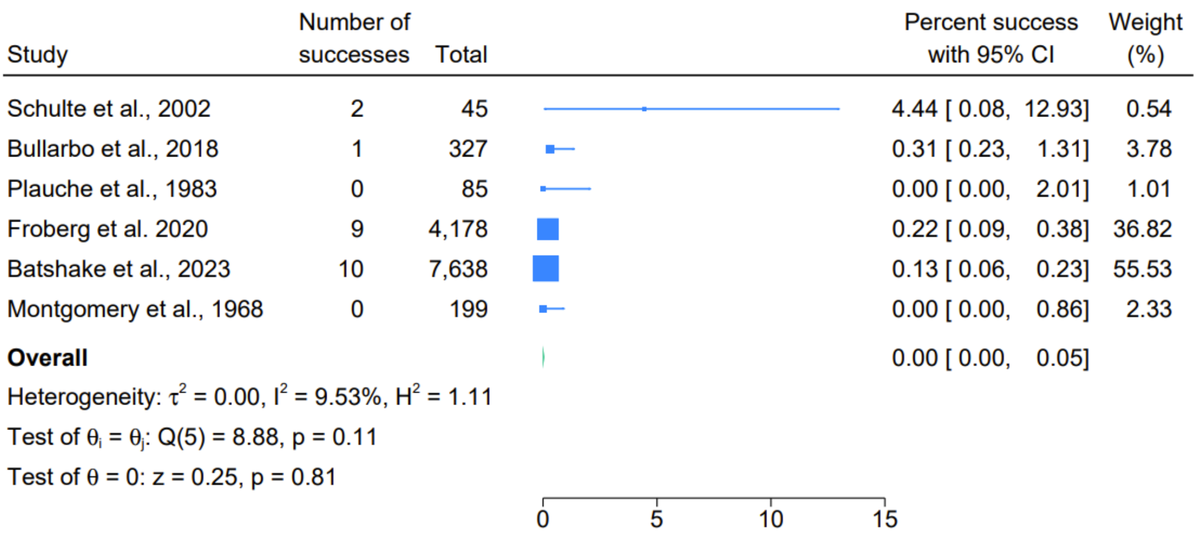

Supplementary Figure 4. Forest plot of the positive predictive value (PPV) of antenatal TB Tuberculin Skin Test (TST)/ Interferon Gamma-Release Assay (IGRA) screening. The number of successes refers to individuals diagnosed with TB disease, and the total represents those who tested positive in the symptom screen. The green diamond represents the estimated overall PPV. The blue boxes show the individual study estimates and the blue lines denote the 95% confidence intervals (CI).

Appendix S6. Additional figures

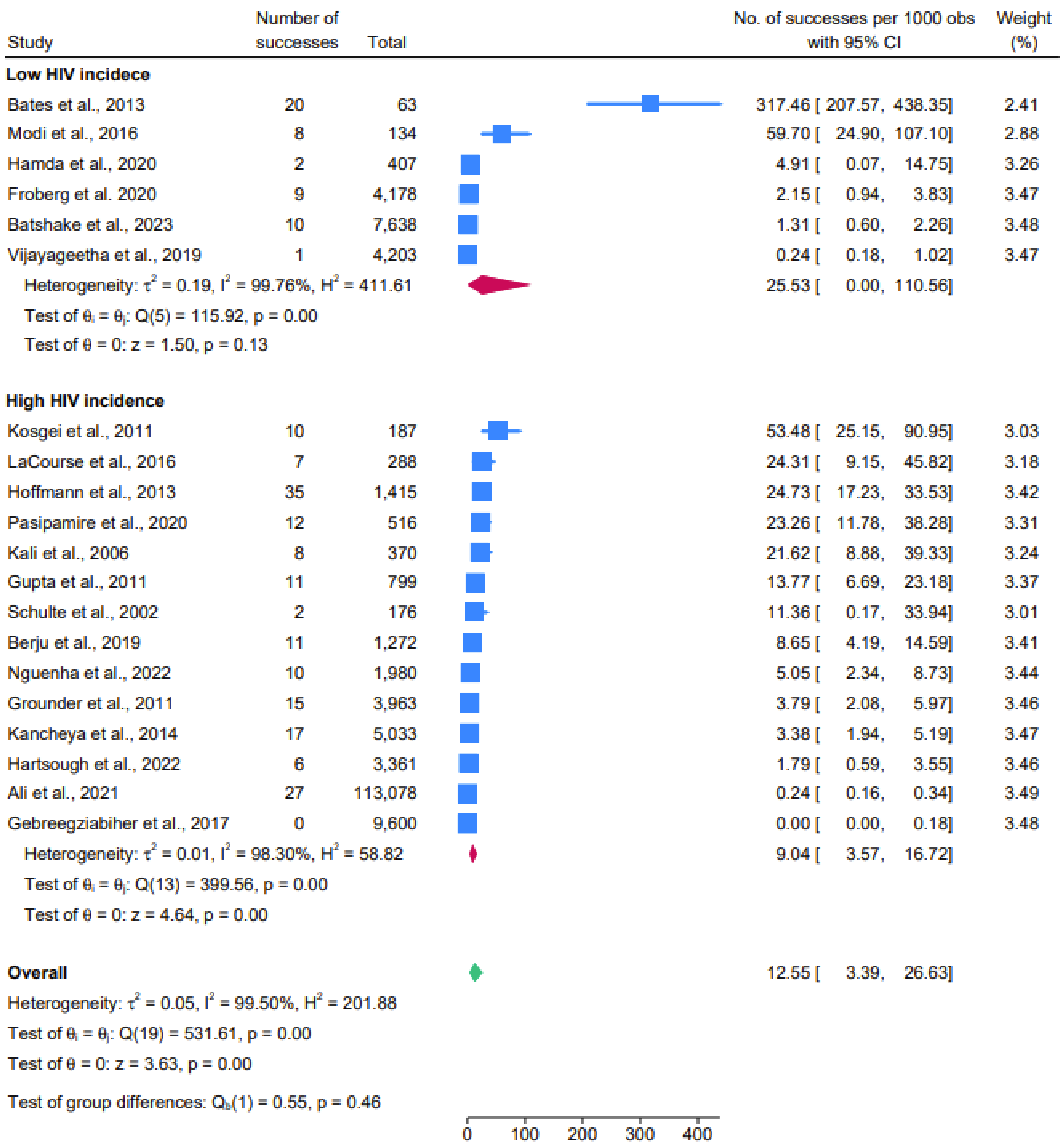

Supplementary Figure 5. Forest plot of the active TB case yield per 1,000 women undergoing antenatal TB screening, stratified by low and high HIV incidence in study population. The number of successes refers to individuals screened as positive, and the total represents the total number of women screened. The red diamonds indicate the estimated group-specific pooled yield, while the green diamond represents the estimated overall yield. The blue boxes show the individual study estimates and the blue lines denote the 95% confidence intervals (CI).
